# Modelling and manufacturing of 3D-printed, patient-specific, and anthropomorphic gastric phantoms: a pilot study

**DOI:** 10.1038/s41598-020-74110-z

**Published:** 2020-11-04

**Authors:** Jinhee Kwon, Joonmyeong Choi, Sangwook Lee, Minkyeong Kim, Yoon Kyung Park, Do Hyun Park, Namkug Kim

**Affiliations:** 1 Department of Convergence Medicine, Asan Medical Institute of Convergence Science and Technology, University of Ulsan College of Medicine, Asan Medical Center, Seoul, 05505 South Korea; 2 ANYMEDI Inc, Seoul, 05505 South Korea; 3 Gastroenterology, University of Ulsan College of Medicine, Asan Medical Center, Seoul, 05505 South Korea; 4 Department of Internal Medicine, University of Ulsan College of Medicine, Asan Medical Center, Seoul, 05505 South Korea

**Keywords:** Gastroenterology, Biomedical engineering, Mechanical engineering

## Abstract

Interventional devices including intragastric balloons are widely used to treat obesity. This study aims to develop 3D-printed, patient-specific, and anthropomorphic gastric phantoms with mechanical properties similar to those of human stomach. Using computed tomography gastrography (CTG) images of three patients, gastric phantoms were modelled through shape registration to align the stomach shapes of three different phases. Shape accuracies of the original gastric models versus the 3D-printed phantoms were compared using landmark distances. The mechanical properties (elongation and tensile strength), number of silicone coatings (0, 2, and 8 times), and specimen hardness (50, 60, and 70 Shore A) of three materials (Agilus, Elastic, and Flexa) were evaluated. Registration accuracy was significantly lower between the arterial and portal phases (3.16 ± 0.80 mm) than that between the portal and delayed phases (8.92 ± 0.96 mm). The mean shape accuracy difference was less than 10 mm. The mean elongations and tensile strengths of the Agilus, Elastic, and Flexa were 264%, 145%, and 146% and 1.14, 1.59, and 2.15 MPa, respectively, and their mechanical properties differed significantly (all *p* < 0.05). Elongation and tensile strength assessments, CTG image registration and 3D printing resulted in highly realistic and patient-specific gastric phantoms with reasonable shape accuracies.

## Introduction

The World Health Organization reported that the prevalence of overweight and obesity have nearly tripled worldwide since 1975^1^. Overweight and obesity have been officially recognized as major risk factors for chronic diseases, such as diabetes, cardiovascular disease, and cancers^2–4^. Various interventional devices including the intragastric balloon (IGB) technique, a cutting-edge non-operative intervention, have been widely used for weight loss of obese patients and treat complications caused by diabetes^5^. However, it is imperative that the balloons be effectively handled in the stomach cavity to minimize migration^6^. For example, after the application of the IGB technique to treat obese patients, potential complications such as balloon perforation and pyloric obstruction were recently reported^6–8^. To date, the optimal inflated volume of IGB has not been evaluated and a fixed volume of inflated IGB has been applied in clinical practice. This fixed and un-adjustable inflated IGB volume may be related to the aforementioned balloon perforation and pyloric obstruction due to its distal migration in some patients. Therefore, repeated evaluations of the IGB technique are necessary to verify its safety prior to its placement in a patient’s stomach. Although several studies of volunteers or obese patients have demonstrated the safety of IGB or identified optimal balloon characteristics^8–10^, no study to date has demonstrated the usability of novel IGB using a patient-specific phantom, which would function as a tool for accelerated virtual ex-vivo study. To enable this evaluation, the current pilot study examined the mechanical properties of the candidate 3D-printing materials for construction of the patient-specific gastric phantom.

An anthropomorphic gastric phantom, which features patient-specific morphology, is required to evaluate IGB performance prior to its placement in a patient’s gastrointestinal tract. In addition, the gastrointestinal phantom used to investigate IGB performance must be fabricated with durable and flexible materials for enabling the simulation of gastric motility. In previous studies including gastric simulators, in vivo and in vitro experiments were performed by replicating the gastric internal tract^9,11–13^. However, in-vivo tests such as animal experiments are difficult to reproduce due to poor repeatability^9,11^. Several studies have fabricated gastric phantoms to evaluate the performance of gastric medical devices. However, these gastric models were not fabricated based on patient-specific images; moreover, it might be difficult to simulate actual conditions because the stomach morphology was not modelled using patient images to implement patient-specific interventions^12,13^.

Three-dimensional printing technology was recently used to create organ phantoms in the medical field^14–16^. In particular, 3D-printed patient-specific phantoms could be used to personalize treatment^14–16^ and simulate the clinical situation to allow medical students to practice difficult procedures. A 3D-printed phantom must be fabricated with patient specific data to achieve anatomical realism^17–21^. Although several studies have captured the general shape of the stomach using triphasic computed tomography (CT) scans^17,18^, image registration techniques might be required to duplicate the shape of the stomach using multiple-phase contrast-enhanced CT scans including peristaltic motions. The properties of materials used for 3D printing have not been thoroughly studied for the fabrication of anthropomorphic gastric phantoms. Therefore, this study aimed to fabricate 3D-printed anthropomorphic gastric phantoms that incorporated patient-specific morphology based on CT images and suitable materials with appropriate tensile strength and elongation properties.

## Results

**Figure 1:**
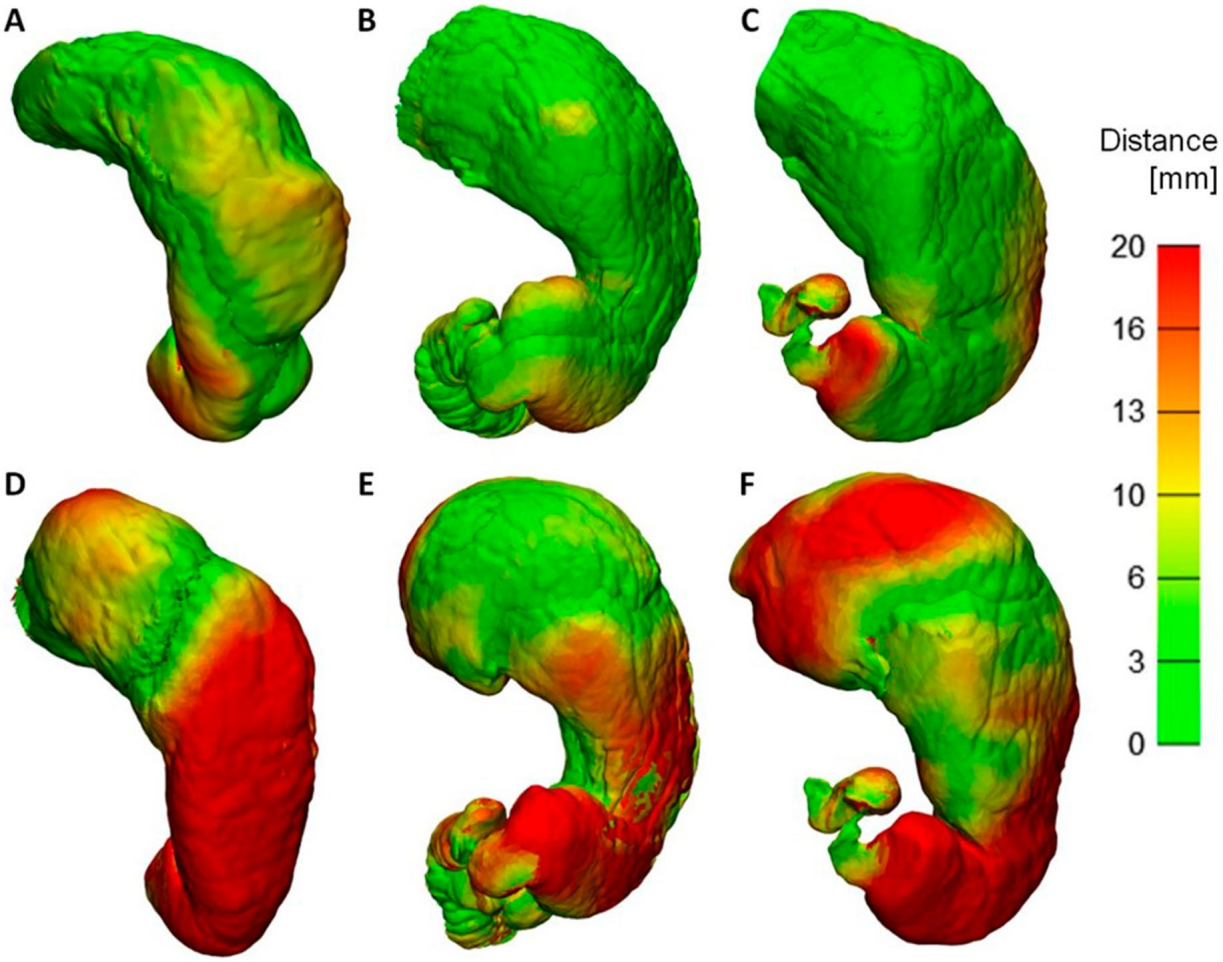
Distance maps comparing the stomach surfaces of the two phases after registration (**A**)–(**C**): arterial and portal phases; (**D**)–(**F**): portal and delayed phases). Each column represents one patient (**A**), (**D**): Patient 1; (**B**), (**E**): Patient 2; (**C**), (**F**): Patient 3).

Figure 1 shows the distance maps created after registration from the alignment of two stomach models that were generated from different CT scan images from the same patient to extract more information about the stomach’s shape. The color map presents the morphological discrepancies among the CT phases. A spatial difference map with colored distributions was used to compare the two phases. The distance maps (Fig. 1A–C) were evaluated by subtracting the portal phase from the arterial phase. In contrast, Fig. 1D–F compare the distance maps of the portal and delayed phases. Table 1 shows that all three mean distances of the discrepancies between the portal and arterial phases (3.9, 2.3, and 3.3 mm) were smaller than those between the portal and delayed phases (10.0, 8.5, and 8.2 mm). The smaller morphological discrepancies between the portal and arterial phases was caused by the structural similarities within the images of the portal and arterial phases based on the degree of distention.

**Table 1:** Measurements taken on the distance maps after registration.

	AP (mm)	DP (mm)
	Mean (IQR)	Mean (IQR)
Patient 1	3.9 (0.9–5.8)	10.0 (2.3–19.8)
Patient 2	2.3 (0.0–3.2)	8.5 (2.0–14.7)
Patient 3	3.3 (0.0–4.8)	8.2 (1.4–14.4)

*IQR* interquartile range, *AP* discrepancy of distances between two stomach morphologies (arterial and portal phases), *DP* discrepancy of distances between two stomach morphologies (delayed and portal phases).

**Figure 2:**
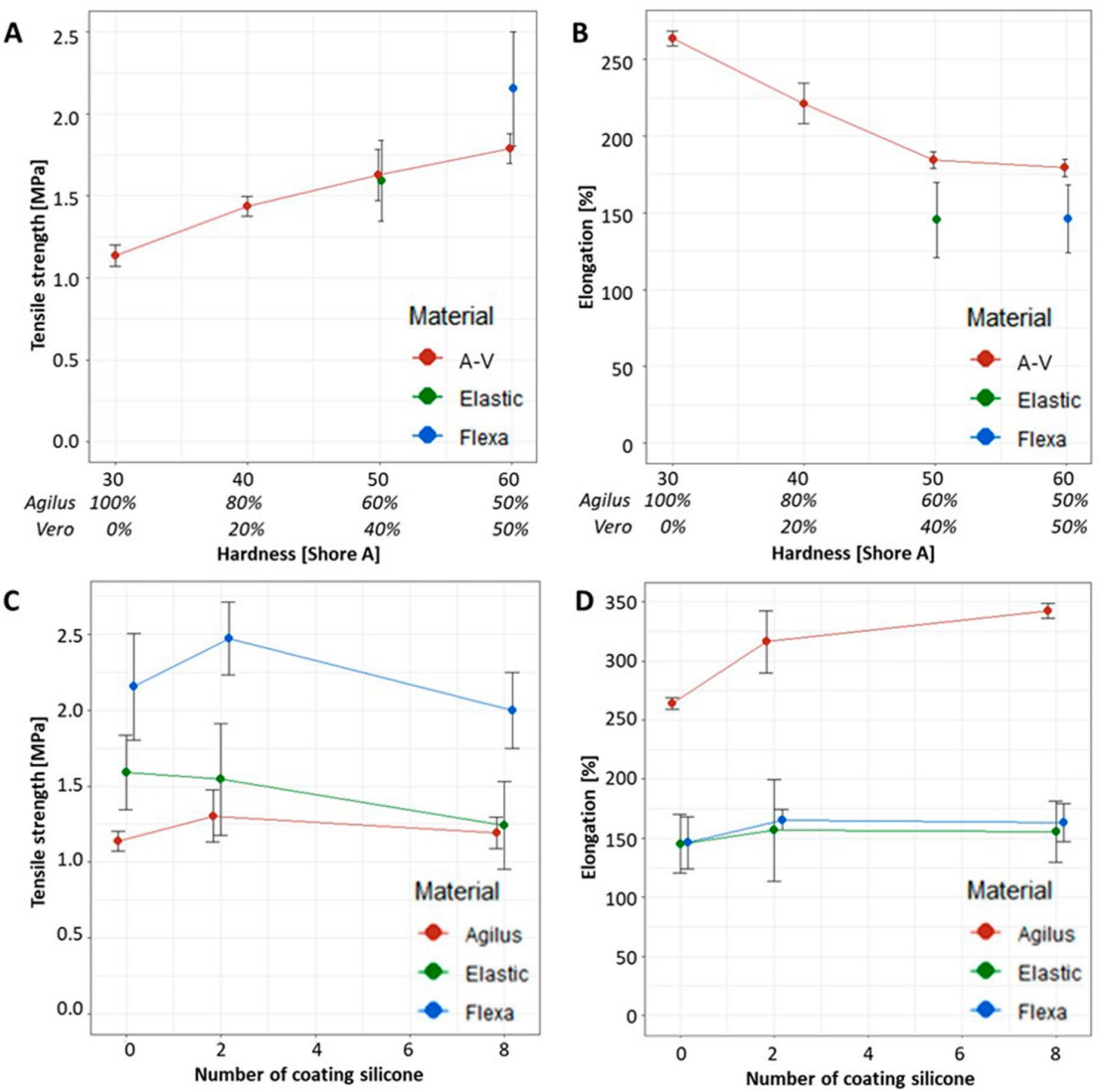
The mechanical properties of specimens with various hardness degrees and numbers of silicone coatings. Four different composites of the Agilus with Vero (A-V) were printed to control for hardness value of 30, 40, 50, 60 Shore A (**A**): tensile strength [MPa]; (**B**): elongation [%]). The mechanical properties are presented according to the number of coatings for all three materials (**C**): tensile strength [MPa]; (**D**): elongation [%]). The plots were drawn using R packages dplyr and ggplot2.

We measured the tensile properties of each specimen according to material type, the number of silicone coatings, and hardness (Table 2, Fig. 2). Table 2 lists the tensile strengths and elongations of three materials according to the number of silicone coatings (0, 2, or 8) and the associated uncertainties. The tensile strengths and elongations of the three materials were significantly different when the number of silicone coatings was the same based on the Kruskal–Wallis test (*p* < 0.05) except for the tensile strength results between the Agilus and Elastic (2 times, *p* = 0.31; 8 times, *p* = 0.84) based on Wilcoxon signed-rank test. Figure 2A,B show the trends of increases in tensile strength and decreases in elongation of the specimens with an increase in material hardness derived from the vendors’ reference measurements. Ratios of Vero in the Agilus and Vero combinations from 0 to 50 have positive association with hardness values. As hardness increased, tensile strength increased and elongation decreased. These results might not show significant differences in tensile strength at the same hardness condition based on the Wilcoxon signed-rank test (A-V vs. Elastic at 50 Shore A, *p* = 0.86; A-V vs. Flexa at 60 Shore A, *p* = 0.15), whereas significant differences in elongation were seen in the same hardness condition based on the Wilcoxon singed-rank test (A-V vs. Elastic at 50 Shore A, *p* < 0.05; A-V vs. Flexa at 60 Shore A, *p* < 0.05). Figure 2C,D presented tensile strengths and elongation from three materials in accordance with the number of silicone coatings (0, 2, 8 times). The tensile strength of the materials did not change significantly with the number of silicone coatings (Fig. 2C). Figure 2D shows that the elongation of only the Agilus increased significantly with the number of silicone coatings, whereas those of the Elastic and Flexa did not differ significantly with different number of silicone coatings based on the Mann–Whitney U test (Agilus, *p* < 0.05; Flexa, *p* = 0.13; Elastic, *p* = 0.65).

**Table 2:** Measurement of mechanical properties including tensile strength and elongation of three materials by the number of silicone coatings.

NCS	Agilus mean (IQR)	u	Elastic mean (IQR)	u	Flexa mean (IQR)	u
**Tensile strength (MPa)**						
0	1.14 (1.12–1.18)*	0.03	1.59 (1.50–1.77)**	0.11	2.15 (2.14–2.29)***	0.16
2	1.30 (1.17–1.44)*	0.08	1.54 (1.39–1.81)	0.17	2.47 (2.26–2.62)***	0.11
8	1.19 (1.11–1.26)*	0.05	1.24 (1.00–1.30)	0.13	2.00 (1.88–2.18)***	0.11
**Elongation (%)**						
0	264 (260–266)*	2.15	145 (142–159)**	11.05	146 (149–156)***	9.89
2	316 (296–334)*	11.69	157 (146–182)**	19.17	165 (159–169)***	3.97
8	343 (336–347)*	2.79	156 (133–165)**	11.66	163 (157–175)***	7.32

*IQR* interquartile range, *NCS* the number of silicone coatings on the three-dimensionally printed specimen, *u* uncertainty.

*p < 0.05 on Kruskal–Wallis test to compare the Agilus, Elastic, and Flexa.

**p < *0.05* by Wilcoxon signed-rank test to compare the Agilus and Elastic.

***p < 0.05 by Wilcoxon signed-rank test to compare the Agilus and Felxa.

**Figure 3:**
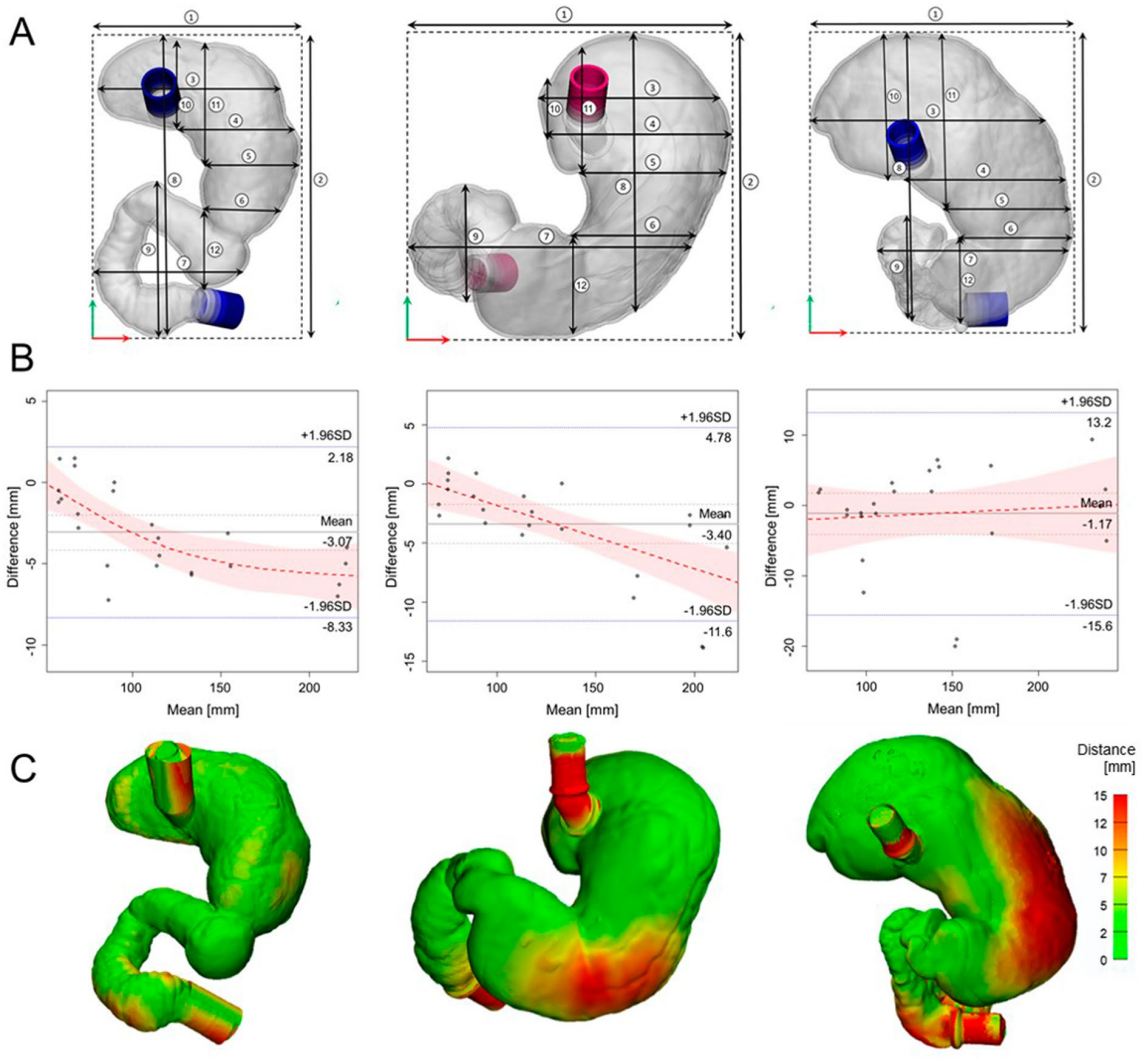
Comparison of STL models and 3D-printed models with (**A**) twelve lengths based on landmarks, (**B**) the Bland–Altman plots present differences in mean between STL and 3D-printed models from the three patients. STL, stereolithography; 3D, three-dimensional (R packages: blandr, ggplot2). (**C**) The distance maps with color legend show overall discrepency between surfaces of STL and 3D-printed models.

We compared the stereolithography (STL) and 3D-printed models to estimate their volumes, surface and lengths (Table 3). The volumes of the 3D-printed models (Patient 1, 880 mL; 2, 1579 mL; 3, 1697 mL), which were modelled by CT scanning of phantoms, were similar to those of STL models (Patient 1, 868 mL; 2, 1504 mL; 3, 1683 mL) from CT scan of the corresponding patients. With regard to the length measurement, twelve lengths were defined in the STL models and 3D-printed models including six horizontal and six vertical distances (Fig. 3A). Figure 3B shows the Bland–Altman plots that presented the differences between the STL and 3D-printed models per patient (mean ± 95% limits of agreement: − 3.07 ± 1.07, − 3.4 ± 1.66, and − 1.17 ± 2.94 mm). In addition, the blue lines present approximate lower and upper 95% confidence intervals for average difference between STL and 3D-printed models (patient 1: − 8.33 – 2.18, patient 2: − 11.57 – 4.78, patient 3: − 15.6 – 13.2). Figure 3C shows the distance map which estimates the overall differences between the surfaces of STL and 3D-printed models.

**Table 3:** The differences between 3D-modelled phantom and 3D-printed phantom.

Volume (mL)	Surface area (mm^2^)	MP (mm)	Mean (IQR)
**Patient 1**			
3D-modelled phantom	$0.87{\times 10}^{3}$	$73.2{\times 10}^{3}$	1.63 (1.25–2.05)
3D-printed phantom	$0.88\times {10}^{3}$	$73.9\times {10}^{3}$	
**Patient 2**			
3D-modelled phantom	$1.51\times {10}^{3}$	$93.9\times {10}^{3}$	3.78 (3.65–5.25)
3D-printed phantom	$1.57\times {10}^{3}$	$95.5\times {10}^{3}$	
**Patient 3**			
3D-modelled phantom	$1.68\times {10}^{3}$	$102.9\times {10}^{3}$	4.47 (3.60–5.73)
3D-printed phantom	$1.69\times {10}^{3}$	$103.3\times {10}^{3}$	

*IQR* interquartile range, *3D-modelled phantom* the 3D-models were built using CT image of patients, *3D-printed phantom* the 3D-models were built using CT image of 3D-printed phantom, *MP* distance discrepancy between two stomach morphologies (3D-modelled and 3D-printed phantoms).

## Discussion

**Figure 4:**
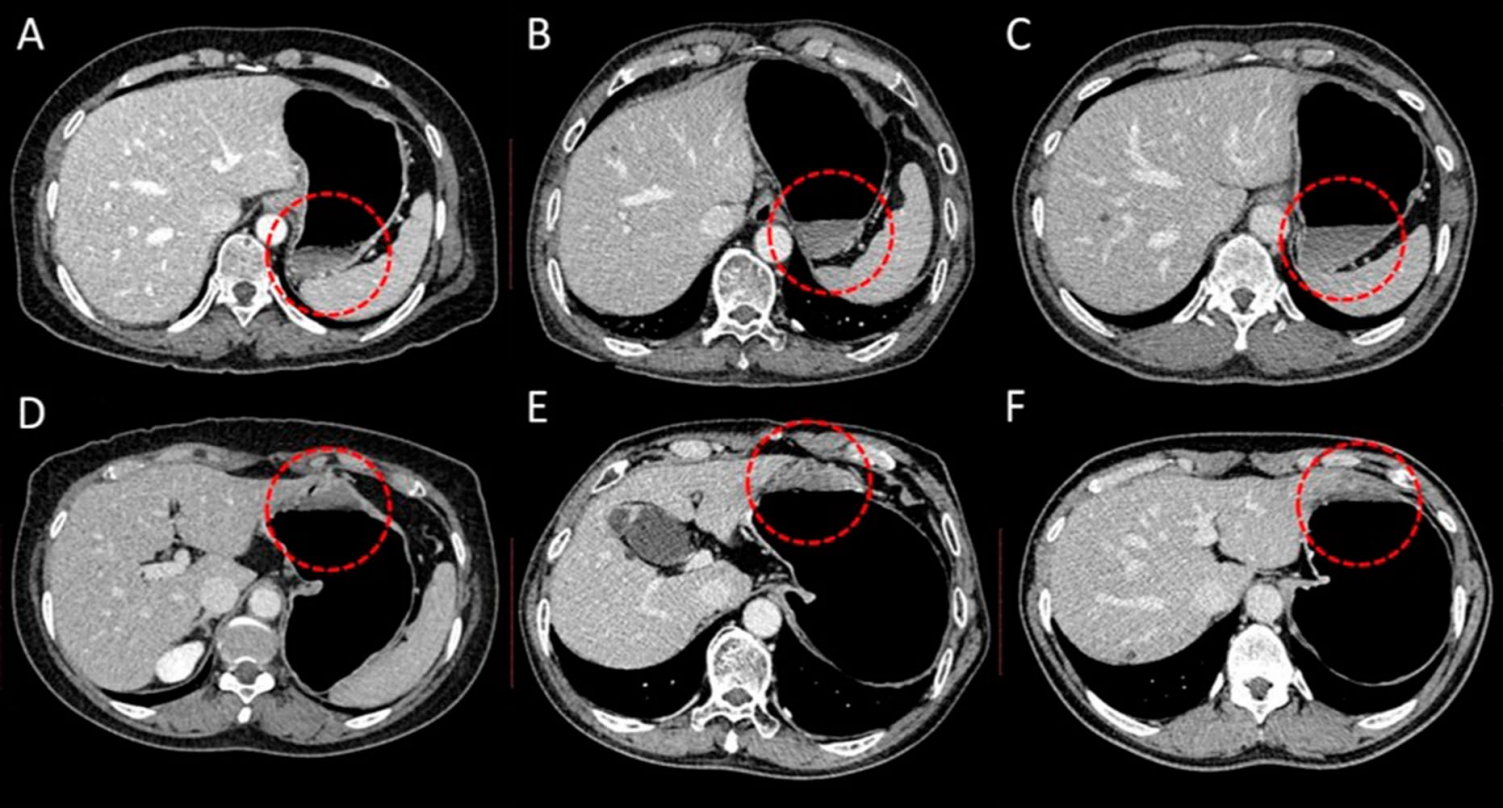
Three patients underwent computed tomographic gastrography (CTG) in the supine (**A)**–(**C**) and prone (**D**)–(**F**) positions. Each column represents one patient (**A**), (**D**): Patient 1; (**B**), (**E**): Patient 2; (**C**), (**F**): Patient 3. The residual fluid filling some portion of the gastric cavity depending on the scanning position is outlined in red.

Several studies have studied and fabricated gastrotomy simulators using computed tomography gastrography (CTG) images to show their efficacy and realism for useful training simulations^17,18^. However, it might be difficult to represent the gastric morphology using only a CTG image due to the nonrigid and constantly moving nature of the organ. Because the portal phase of CTG was collected with the patient in the supine position, the image could not be acquired due to air bubbles in the upper gastric portion and fluid in the lower gastric region (Fig. 4). The duodenum was segmented from the arterial phase scan with the patient in the left posterior oblique position to identify the antrum. The distance discrepancies, represented by the mean distances in Fig. 1, were probably due to the quantification of a previous clinical observations made by Kim et al.^22^ relating to structural similarity within image (portal and arterial) phases based on the degree of distention. In particular, Kim et al.^22^ reported that the portal (supine) and arterial (left posterior oblique) phase images did not show statistically significant differences in mean gastric distention; this finding is in contrast with the prone position (delay), which had a significantly increased score for the upper gastric portion compared to the supine and left posterior oblique positions*.* Because the delayed phase was collected with the patient in the prone position, this image is regularly used in the fundus evaluation, where the upper gastric portion stands out in the image^22^. Therefore, the gastric model is designed by registration of the arterial, portal, and delayed phases to capture the overall morphology considering the morphologic differences among them. The registration of the portal and delayed phases was more obvious than that of the portal and delayed phases. The overall distance was less than 3.9 mm between the portal and arterial phases and 10.0 mm between the portal and delayed phases. Through the registration among the stomachs from each phase, the realistic and patient-specific stomach morphology could be captured.

**Figure 5:**
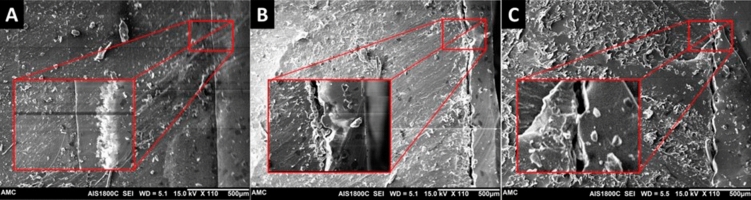
Scanning electron microscopy images of the surface layer used to investigate the integrity of the interfaces among the printed materials ((**A**), Agilus; (**B**), Elastic; (**C**), Flexa) and the silicone coating.

We also performed quantitative mechanical testing to evaluate the candidate materials for the gastric phantom, which requires flexibility and durability, with various hardness conditions and the number of silicone coatings. Based on the tensile test results of the previous study, the range of elongation for fundus tissue was 241% (SD: 83%), whereas the range of tensile strength for fundus tissue was 0.23 MPa (SD: 0.06 MPa)^23^. Compared with results for the human stomach, the elongation of the Agilus showed the most similar ranges (0, 2, and 8 silicone coatings: 264, 316, and 343%, respectively), whereas none of the candidate materials demonstrated similar tensile strength ranges. In the tensile strength test, the Agilus was the most flexible of the three rubber-like materials since it had the lowest Shore hardness and greatest elongation. The elongation of the Agilus only increased with the number of silicone coatings. Therefore, the interface between the printed material and silicone coating was investigated using scanning electron microscopy. As shown in Fig. 5, we measured the distance of the interface between specimens and silicone coating 20 times. The distances of the interfaces with the Agilus, Flexa, and Elastic were 3.85 μm (SD: 0.81 μm), 29.78 μm (SD: 9.96 μm), and 20.98 μm (SD: 7.65 μm), respectively. In particular, the silicone coating was consistently and firmly laminated with the surface of the Agilus specimen, whereas the silicone was inconsistently and weakly laminated with the surfaces of the Flexa and Elastic specimens. Due to the high viscosity of the silicone, it might not have evenly coated the surfaces of the Flexa and Elastic specimens (Fig. 5). Hence, we will further investigate the various silicone coating methods in detail to improve the mechanical properties of the gastric phantom.

There are several limitations to this study. First, it included only three patients, a sample size that might be insufficient to demonstrate the reproducibility of the gastric phantom manufacturing process. Accordingly, we plan to enroll a larger number of obese patients in a future study. Second, the threshold value for determining the gastric cavity was not quantitatively defined in a previous study so that we assumed the imaging threshold as specified range from − 1024 to − 500 HU; rather, we must determine the adequate threshold in a future study. Third, limited trials were used to evaluate the mechanical properties of these materials. Therefore, in further studies, the candidate materials must be precisely evaluated and the various mechanical properties improved using post-processing materials such as silicone coatings. Fourth, the IGB evaluation using the 3D-printed phantom was not the subject of this study; rather, the design and printing methodology are presented along with the mechanical properties of the candidate materials. Therefore, further studies of the 3D-printed phantom for the IGB evaluation may be required to clarify the role of this novel device in clinical training or practice. Finally, the shape accuracy error of the phantom was relatively large considering the printing accuracy. These errors were probably caused by the hollow structures of the stomach, deformation of the soft material^24^, and measurement errors. For further studies, we must develop a more accurate gastric phantom with better design, printing, and measurement variables.

## Conclusion

In this study, registration of three phases of CTG images was performed to determine the overall stomach morphology, whereas shape accuracy was evaluated to fabricate anthropomorphic and patient-specific gastric phantoms. Mechanical properties including elongation and tensile strength were evaluated using various number of silicone coatings to produce a more realistic phantom. Therefore, 3D-printed and patient-specific gastric phantoms with mechanical properties similar to those of the human stomach were created that could be used to simulate various interventional devices including IGB.

## Methods

The Institutional Review Board for Human Investigations of Asan Medical Center (IRB no. 2019–1223) approved this retrospective study and waived the need for informed consent accordingly for the acquisition of imaging data on CT scans. The imaging data were de-identified in accordance with the Health Insurance Portability and Accountability Act of 1996.

### CT image acquisition

This study retrospectively examined CTG images of three patients taken at Asan Medical Center. The patients fasted at least 8 h prior to undergoing CTG with a standard protocol of 100 kVp and slice thickness of 1.0 mm. Buscopan 10 mg (Boehringer Ingelheim, South Korea, Seoul) was intravenously injected to stall bowel peristalsis. To achieve gastric distension during CTG, patients ingested 6 g of effervescent granules (Taejeon Pharmaceuticals, Kyungki-Do, South Korea) in 10 mL of water. Triphasic dynamic CT scans including arterial, portal, and delayed phase images were used to evaluate gastroduodenal strictures along with partial shape. The arterial phase was collected with the patient in the left posterior oblique position (start of delay: 30 s), portal phase was collected with the patient in the supine position (start of delay: 72 s), and delayed phase was taken with the patient in the prone (start of delay: 150 s) position^22^. We also performed a 3D CT scan of the 3D-printed phantoms using the standard protocol of 100 kVp and slice thickness of 0.6 mm.

### Imaging processing, registration, and modelling

**Figure 6:**
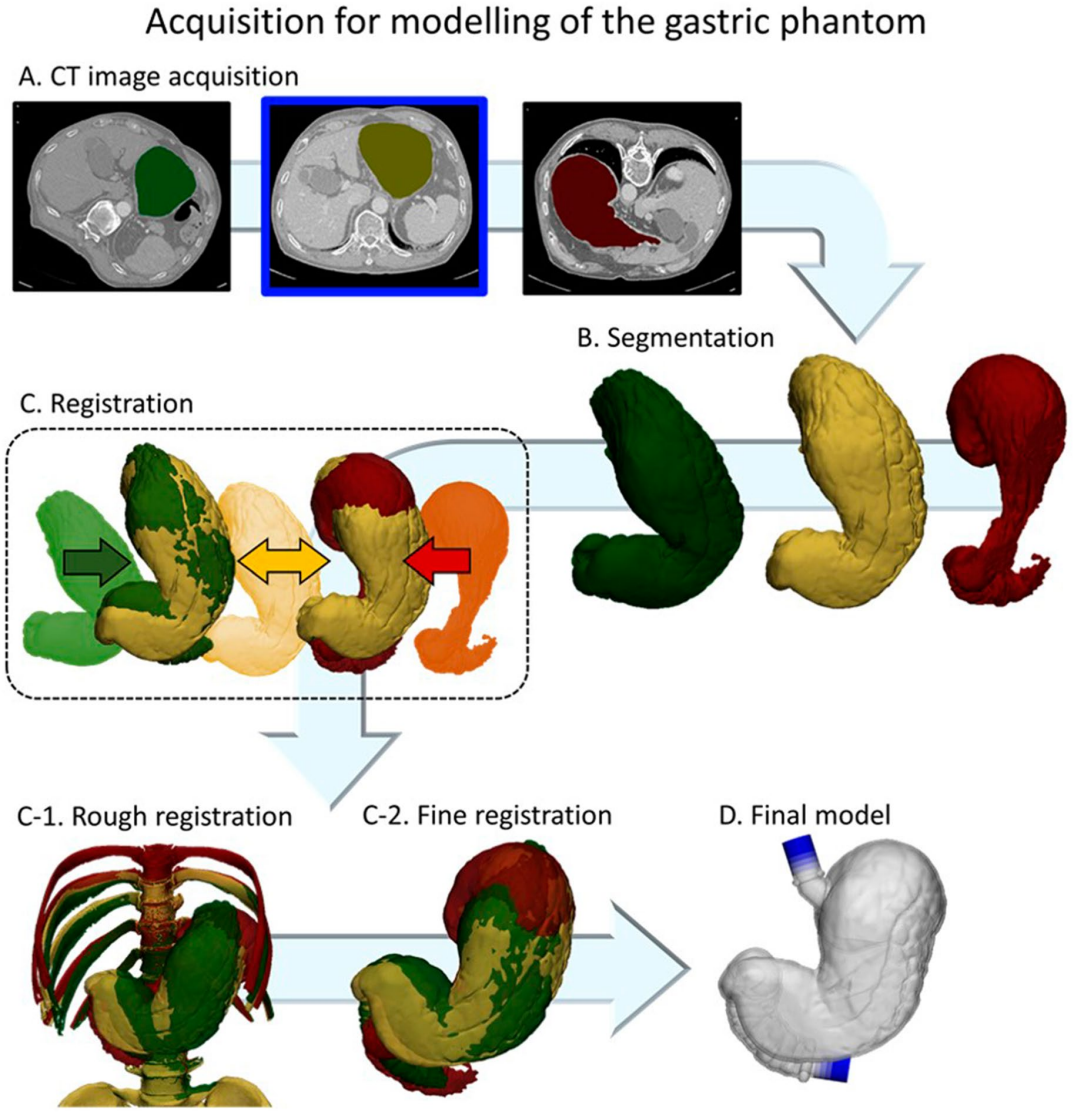
The overall procedure from computed tomography acquisition to modelling the gastric phantom. (**A**) Three phases of CTG scans collected with the patient in different positions including the arterial phase in the left posterior oblique (green), portal phase in the supine (yellow), and delayed phase in the prone (red) positions. The portal phase was used as reference (blue outline). (**B**) The stomach was segmented from the images by the defined thresholds. (**C**) Registration was performed as follows: rough registration based on supine alignment (C-1) and fine registration based on surface registration algorithm between the stomach images (C-2). (**D**) The final stomach model with an attached inlet and outlet.

Digital Imaging and Communication in Medicine images (DICOM) of the CTG scans were imported into Mimics Research 17.0 software (Materialise, Leuven, Belgium) to directly segment objects including the stomach body, duodenum, and spine, which were then exported into an STL file with triangular tessellation. The Hounsfield unit (HU) threshold for gastrointestinal tract including the stomach body and duodenum was determined by thresholding from -1024 to -500 HU and the spinal bone by thresholding at above 1000 HU. We also performed the registration of the three phases of the CTG scans to reliably catch overall stomach morphology from each phase using 3-matic 9.0 software (Materialise). Rigid registration was initially performed to align the nearby spine from three phases by manually choosing the spatial correspondence of each spine using the N-point registration algorithm in 3-matic 9.0. In this step, the portal phase with patient position in supine position was used as a reference to align the arterial and delayed phases because it is between the arterial and delayed phases and could have features similar to other phases. Next, fine registration was automatically performed to minimize the overlapping local surfaces of each stomach model using the surface registered algorithm in 3-matic 9.0 (Fig. 6). The difference between the overlapped volume and interfaced volume was analyzed to investigate the differences between stomach models segmented from the CT images. The distance map between the stomach models from the three different phases was used for the registration assessment (Fig. 1).

### 3D printing and mechanical evaluation

**Figure 7:**
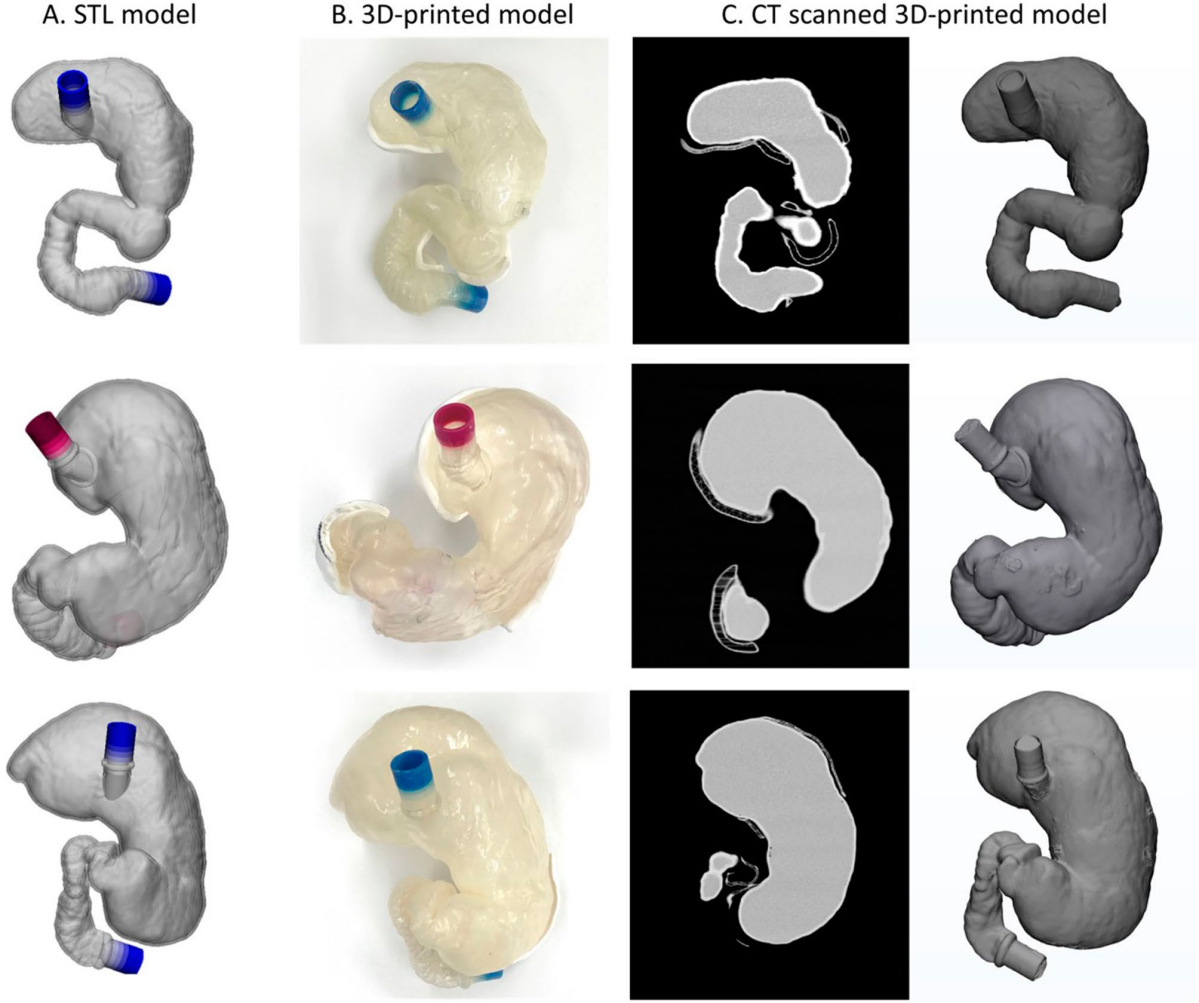
Based on the (**A**) 3D stereolithographic (STL) gastric models (**A**), derived from patient CT images, (**B**) the phantom were fabricated using a 3D printer. (**C**) CT scans of the gastric phantoms enabled comparison with the corresponding STL models.

A wall thickness of 2.5 mm was determined by offsetting of the final gastric model to mimic the wall thickness of the human stomach using 3-matic 9.0 (Materialise) with the offset tool. After the inner layer was uniformly offset, the outer layer was created with Wrapping and Smoothing tools to represent the flattened layer using 3-matic 9.0 (Materialise). This process could lead to the non-uniformity, but the mean thickness of the wall converges to 2.5 mm. This 3D STL models in Fig. 7-A were saved in G-code format and exported to a 3D printer (Objet500 Connex3; Stratasys Ltd., Rehovot, Israel). The phantom was made of 100% rubber-like material (Agilus Transparent; Stratasys Ltd.) with a silicone coating (MED6-6606; NuSil, CA, USA) to enhance its durability and flexibility (Fig. 7-B). The silicone coating was applied using an in-house rotator machine to ensure uniformity. In addition, the CT scanned 3D-printed phantom images were imported into the Mimics Research 17.0 software (Materialise) to segment the phantoms (Fig. 7-C). The printing accuracy was assessed by comparing volumes of the STL model and the 3D printed phantom calculated by 3-matic 9.0 (Materialise). During the CT evaluation, the 3D printed phantoms are completely filled with saline. To test the tensile strength of the rubber-like elastomer, ASTM D412 type C, the most common standard specimen model designed to measure the tensile strength of three 3D printed materials, was used^25^. Each specimen was equivalently adjusted to use the same ASTM type and printing direction, and its hardness and the number of silicone coatings on the specimen were controlled with several parameters. Based on the commercial data sheets, we reported the hardness values of all specimens. The unit of each hardness was set as Shore A based on the shore hardness scales. The specimen consisted of five standard dog-bone-shaped pieces to compare the mechanical properties of the different materials printed from the three different 3D printers (material/equipment; Elastic/Form 2, Formlabs Inc., MA, USA; Flexa 693/XFAB, DWS Inc., Meccanica, Italy; Agilus Translucent, Vero Magenta/Objet500, Stratasys, Ltd., MN, USA). These printers were selected for their ability to print rubber-like material along with their high resolution accuracy and large work area. The Form 2 and XFAB 3D printers employ technology based on laser stereolithography, whereas the Objet500 uses PolyJet printing. Among them, the Objet500 can control the flexibility by blending photopolymer resins Agilus and Vero from rubber to rigid. To vary object hardness and create multi-material versatility, we selected the specific ratio between the Agilus and Vero. Specimens fabricated with Agilus and Vero combinations from 100:0 to 50:50 were used to simulate specific hardness values including various ranges of Shore A, which were fabricated by the Objet500. The differences in the specimen post-fabrication by the number of silicone coatings (0, 2, and 8 times) were evaluated. We also measured the differences among all specimens five times in terms of the silicone coatings using ythe Vernier scale (See Supplementary Fig. S1 online). All specimens were mounted and tested in an electromechanical testing machine (Instron 5882; INSTRON, MA, USA) equipped with a 5-kN load cell. The thickness of all rubber-like specimens was set to 2.5 mm and a constant extension rate of 50 mm/min was applied until the specimens failed^26^. The mechanical properties including tensile strength (MPa) and elongation (%) were assessed using the tensile test according to references of previous studies that performed tensile tests to demonstrate the mechanical properties of stomach tissues^23,27^.

### Statistical evaluation

The descriptive statistics of the shape accuracy of the 3D printing and tensile strength test were evaluated. Continuous variables are expressed as mean and interquartile range. Because the sample we used was nonparametric group, Mann–Whitney U test was used to compare the differences along with the number of silicone while Kruskal–Wallis test was used to compare the differences along with material type, followed by post-hoc analysis with the Wilcoxon signed-rank test. We calculated the uncertainty in measurement to investigate the range of possible values about the true value of the measurement results. Two-tailed *p*-values less than 0.05 were considered significant. The Bland–Altman plot was used to evaluate the shape accuracies of the STL and 3D-printed models. The differences between the STL and 3D-printed phantoms, 95% limits of agreement and 95% confidence intervals were calculated. Based on the same viewpoint image of the stomach, the twelve landmarks of the STL models and 3D printed phantoms were measured two times with 3-matic 9.0 (Materialise). The twelve landmarks were described as the longest both horizontal and vertical lengths of the 2D view (1, 2; Fig. 3) and specific horizontal and vertical distances from the phantom edge to the other edge (3, 4, 5, 6, 7, 8, 9, 10, 11, 12; Fig. 3). All statistical analyses were performed using R version 3.4.2 (R Foundation for Statistical Computing, Vienna, Austria)^28–30^.

## Supplementary information

Supplementary Figure 1.

## Data Availability

The datasets generated during and/or analyzed during the current study are not publicly available due to legal issues but are available from the corresponding author on reasonable request.
