# Supplementary figures and images for: Modelling and manufacturing of 3D-printed, patient-specific, and anthropomorphic gastric phantoms: a pilot study
Source: Sci Rep. 2020 Nov 4;10:18976. doi: 10.1038/s41598-020-74110-z (PMC7643145; doi:10.1038/s41598-020-74110-z)

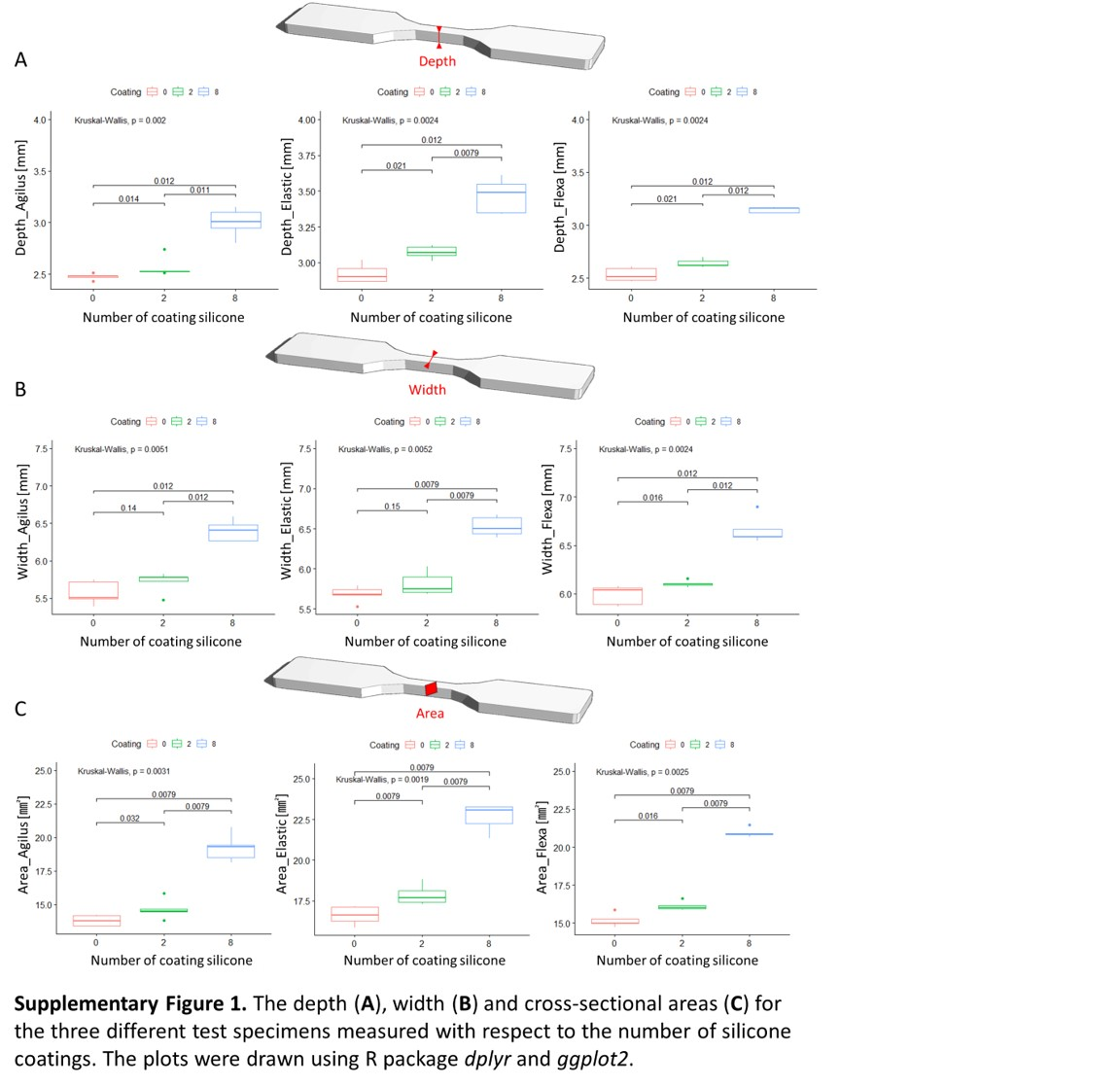

Supplement: Supplementary file 1 — Supplementary Figure 1. [file 41598_2020_74110_MOESM1_ESM.png]
